# Effects of decades of physical driving on body movement and motion sickness during virtual driving

**DOI:** 10.1371/journal.pone.0187120

**Published:** 2017-11-09

**Authors:** Thomas A. Stoffregen, Chih-Hui Chang, Fu-Chen Chen, Wei-Jhong Zeng

**Affiliations:** 1 School of Kinesiology, Affordance Perception-Action Laboratory, University of Minnesota, Minneapolis, Minnesota, United States of America; 2 Department of Physical Education, National Kaohsiung Normal University, Kaohsiung, Taiwan; Tokai University, JAPAN

## Abstract

We investigated relations between experience driving physical automobiles and motion sickness during the driving of virtual automobiles. Middle-aged individuals drove a virtual automobile in a driving video game. Drivers were individuals who had possessed a driver’s license for approximately 30 years, and who drove regularly, while non-drivers were individuals who had never held a driver’s license, or who had not driven for more than 15 years. During virtual driving, we monitored movement of the head and torso. During virtual driving, drivers became motion sick more rapidly than non-drivers, but the incidence and severity of motion sickness did not differ as a function of driving experience. Patterns of movement during virtual driving differed as a function of driving experience. Separately, movement differed between participants who later became motion sick and those who did not. Most importantly, physical driving experience influenced patterns of postural activity that preceded motion sickness during virtual driving. The results are consistent with the postural instability theory of motion sickness, and help to illuminate relations between the control of physical and virtual vehicles.

## Introduction

Motion sickness is an age-old problem, one that recently has been exacerbated by interactive technologies, including simulated and virtual vehicles. One persistent problem is our inability to predict the occurrence of motion sickness in individuals, that is, individual susceptibility. When exposed to a given motion situation, motion sickness occurs in some individuals, but not in others. What accounts for these differing individual responses? One factor that is known to be important to individual susceptibility is individual experience. For example, with continued exposure to stimulus motion, the malady naturally fades. Seasickness may be the best example: sea travelers who are initially sick gradually recover, even though the ship continues to move [1]. It has been suggested that experience influences susceptibility among users of interactive technologies, in general, and video games, in particular [2]. Experience clearly matters, that is, experience leads to changes in the individual that affect susceptibility. The question is, what are these changes? What is the difference between an individual who has had certain experiences and one who has not, and how do these differences relate to motion sickness susceptibility?

These questions relate to broader issues of the etiology of motion sickness, that is, to the causality of motion sickness, in general. A prominent and venerable view of motion sickness etiology is the intersensory conflict theory, which is based upon the hypothesis that experience (i.e., perceptual-motor interactions with the environment) gives rise to internal expectations (sometimes referred to as an internal model) about multisensory patterns that should occur in any given situation [3,4]. The focus on hypothetical expectations derived from experience suggests that a strong test of this theory might be found in situations involving extensive experience with a particular type of interaction with the environment. Driving offers such a situation: Individuals commonly drive for many decades, so that in experienced drivers, hypothetical internal models should be highly robust.

In the intersensory conflict theory of motion sickness etiology, hypothetical experience-based expectations are related to motion sickness susceptibility, but they also have the function of guiding perceptual-motor interactions with the environment [3,5]. That is, experience-based expectations should simultaneously influence motion sickness susceptibility and the details of movement in perceptual-motor interactions with the environment.

### Experience and vehicle simulation

Contemporary driving simulators are associated with the risk of motion sickness. Sickness can occur in any age group, but there is some suggestion that the risk of motion sickness in driving simulators may increase with age [6]. In existing research, however, chronological age has been confounded with participants’ experience driving physical automobiles; that is, older drivers have more driving experience than younger drivers. A similar problem obtains with research on flight simulation. Some studies have reported that the severity of motion sickness in a flight simulator was positively correlated with the amount of time that participants had spent flying the corresponding physical aircraft [7–9]. However, only one study has reported both chronological age and flying experience: Webb et al. [10], reported mean age 24.8 years and 51.1 years, respectively, for less experienced Student pilots and more experienced Instructor pilots. An important limitation of all of these studies is that pilot experience was confounded with chronological age: In each study, more experienced pilots were chronologically older, while less experienced pilots were chronologically younger. Susceptibility to motion sickness varies across the lifespan, but studies reporting this effect have not taken into account age-related variations in experience operating vehicles [11].

In many countries nearly all adults are licensed drivers, such that separation of chronological age and driving experience is impractical. There are, however, countries in which adults with and without driver’s licenses are both common. In these countries, chronological age can be separated from physical driving experience by comparing adults with and without driver’s licenses. Chang et al. [12] compared young adults with and without driver’s licenses. Participants’ mean age was 24 years, and drivers had, on average, 3.40 years driving experience. Each participant drove a virtual vehicle in a driving video game for up to 40 minutes. The overall incidence of motion sickness was 62.5%. Incidence did not differ between drivers (65%) and non-drivers (60%). Among participants who stated that they were motion sick, the severity of symptoms associated with motion sickness did not differ between drivers and non-drivers. These results suggested that approximately three years of experience driving physical vehicles had no effect on susceptibility to motion sickness while driving a virtual vehicle.

### Experience-based internal expectations?

In physical driving, accelerations (speeding up and slowing down, and turns) give rise to changes in stimulation of (at least) the visual and vestibular systems: Occupants both see and feel accelerations. Because they operate the accelerator, brake, and steering wheel, drivers control the automobile’s acceleration, and so they control these patterns of visual and vestibular stimulation. Within the intersensory conflict theory of motion sickness etiology [3–4], experience driving physical automobiles is hypothesized to create internal expectations about relations between visual and vestibular stimulation during accelerations [13]. These expectations will include the temporal sequence between feedback arising from control actions (pressing pedals, turning the wheel), and the resulting inertial forces that, in turn, alter visual and vestibular stimulation.

Fixed-based driving simulators and virtual vehicles, including driving video games, do not reproduce the inertial forces that accompany physical driving and, therefore, do not reproduce relations between the driver’s control actions and patterns of visual and vestibular stimulation that characterize driving in physical automobiles. For this reason, virtual driving should give rise to strong conflict between actual patterns of intersensory input and any patterns expected from experience with physical driving. People who have never driven a physical automobile should have no expectations about sensory feedback arising from their own actions in relation to patterns of intersensory stimulation during automobile travel. Thus, when driving a virtual vehicle, persons who have never driven a physical automobile should experience less intersensory conflict than persons who have driven physical automobiles. By this logic, the intersensory conflict theory would appear to mandate a prediction that—during virtual driving—motion sickness should be more common among persons with physical driving experience than among persons who have never driven a physical automobile. The strength of this effect should increase with experience: Persons with greater experience driving physical automobiles should have more robust hypothetical internal expectations and, therefore, should be be more strongly effected than persons with less experience, or with none.

People who have never driven nevertheless have extensive experience riding in automobiles, as passengers. Experience as a passenger should give rise to expectations about patterns of intersensory stimulation. We might, then, argue that non-drivers would also experience intersensory conflict in a virtual vehicle; not because of the virtual nature of the vehicle but, rather, because of the novel role of driving. This argument may be tempting to supporters of the intersensory conflict theory, but it is not without problems. The argument would necessarily also apply to people who make the transition from passengers to drivers in physical automobiles, that is, to people learning to drive. In industrialized countries, people experience automobile travel as passengers from the very beginning of life (e.g., infants being brought home from the hospital by car). Thus, most people who enter driver training have more than 15 years of experience as automobile passengers and, therefore, should have very robust, stable expectations about intersensory patterns of stimulation as passengers, but not as drivers. Yet, learning to drive is not associated with reports of motion sickness: Student drivers do not get sick. We might ask about student pilots, but that situation is less relevant, as 1) there are simply fewer individuals involved, 2) few people spend a lot of time as passengers in small aircraft and then transition to pilots (as opposed to learning to fly as novice travelers in small aircraft) and 3) no one goes directly from being an airline passenger to becoming an airline pilot, without intervening experience piloting smaller aircraft.

For drivers, control of the body within an automobile can be anticipatory [14]. Drivers know about their own control actions, and for this reason their adjustments of body orientation can be anticipatory. By contrast, passengers cannot know about quantitative details of the driver’s actions until they happen and, for this reason, passengers’ bodily adjustments must be compensatory. So, learning to drive includes a shift from compensatory control to anticipatory control. As noted above, there is no evidence that this change is nauseogenic. By contrast, the shift from driver to passenger can be nauseogenic, even for experienced travelers [13].

Both physical and virtual driving offer anticipatory control; the difference between physical and virtual driving is in the absence of inertial forces that challenge bodily stability. For drivers, this shift in inertial forces may be the principal difference between physical and virtual vehicles. For non-drivers, the shift from physical vehicles (as passengers) to virtual vehicle (as drivers, in our study) entails a shift from the presence to the absence of inertial forces, but also a shift from compensatory to anticipatory control. Given that (in physical vehicles) the shift from passenger (compensatory) to driver (anticipatory) is not associated with anecdotal reports of sickness, we can conclude that motion sickness is related to the shift from physical to virtual vehicles and, within the intersensory conflict theory, to hypothetical expectations that are related to inertial forces as part of intersensory stimulation.

It is important to note that these arguments are logical rather than quantitative. Quantitative predictions about the nature of hypothetical internal expectations about patterns of intersensory stimulation cannot be formulated. The scientist cannot know, in quantitative detail, the history of an individual’s interactions with the environment and, for this reason, cannot know, in quantitative detail, what patterns of intersensory stimulation might be expected. Hypothetical intersensory conflict is defined as the difference between current and expected patterns of intersensory stimulation [3–4]. Because expectations cannot be known in quantitative detail, the magnitude of the difference between current patterns of intersensory stimulation and those (hypothetically) expected on the basis of past interactions with the environment cannot be known in quantitative detail. For this reason, it is exceedingly difficult to use theories based upon the concept of intersensory conflict to make predictions about motion sickness susceptibility in individuals. This logical problem was pointed out by Stoffregen and Riccio ([15], p. 181).

As noted above, in the study of Chang et al. [12] the incidence and severity of motion sickness during virtual driving did not differ between participants with and without experience driving physical automobiles. This result offers no support for the hypothesis that physical driving experience would give rise to expectations about intersensory stimulation that would be violated during virtual driving. However, supporters of the intersensory conflict theory might point out that the young adults tested by Chang et al., had been driving only a relatively short time (approximately 3 years), and so may have not yet had time to develop robust internal expectations about intersensory stimulation during driving. In the present study, one of our goals was to address this issue by including as participants individuals with many years of experience driving physical automobiles.

### Bodily control as an etiological factor in motion sickness

Riccio and Stoffregen [16] offered a novel theory of motion sickness etiology that does not rely upon the concept of intersensory conflict. They suggested that motion sickness arises from instability in the control of the body (the entire body, or its segments). The most direct prediction of this theory is that that there should differences in movement between persons who experience motion sickness and those who do not, and that these differences should exist before the onset of any subjective symptoms of motion sickness. This prediction has been confirmed in laboratory devices [17–19], virtual environments [20], fixed-base flight simulators [21], video games [14,22–25], and seasickness [26].

Chang et al. [12] monitored the kinematics of the head and torso during virtual driving. Participants were explicitly instructed to discontinue their participation immediately if they began to experience any symptoms of motion sickness, however mild. Accordingly, the kinematic data collected by Chang et al., reflected postural precursors of the subjective symptoms of motion sickness. Analysis of these data revealed that patterns of movement differed between participants who (later) became sick and those who did not (in a statistically significant interaction with the duration of exposure to the driving game). This effect replicated numerous previous studies [14,17,19,22]. In addition, Chang et al. [12] found statistically significant interactions between participants’ experience driving physical automobiles and the incidence of motion sickness during virtual driving. Separately in measures of the spatial magnitude and the temporal dynamics of movement, the patterns of movement that preceded motion sickness differed qualitatively between drivers and non-drivers (see [12], Fig 4).

### Physical driving experience and virtual driving

Experience driving physical automobiles could influence the way individuals interact with virtual automobiles. As noted earlier, drivers’ postural adjustments can be anticipatory, while passengers’ adjustments must be compensatory. People typically are passengers before they become drivers: Before a person learns how to drive, they typically have been riding in automobiles for many years. For this reason, a person who has never learned to drive should be skilled at *compensatory* postural adjustments in response to the forces experienced in automobile travel. By contrast, drivers can learn the skill of *anticipatory* postural adjustments. The difference between anticipatory and compensatory postural adjustments, learned in physical automobiles, may carry over into the driving of virtual automobiles. Such differences could affect both bodily movement and motion sickness during virtual driving. Older adults typically have had more extensive experience in automobiles than younger adults, both as drivers (if they are drivers) and as passengers. Thus, we might expect that any effects of driving experience on responses to virtual automobiles would be greater among older adults. Such an effect would be consistent with reports that older drivers are at greater risk of motion sickness during virtual driving [6].

### The present study

We repeated the method of Chang et al. [12], with one exception. Whereas Chang et al. used young adult participants, in the present study participants were middle-aged. Among middle-aged adults, we asked how the experience of driving physical automobiles might affect body movement and motion sickness in when driving a virtual automobile. In Taiwan, the minimum age at which persons may obtain a driver’s license is 18 years. Among persons aged 18 and above, fewer than 70% actually hold a driver’s license, thus making it relatively easy for us to recruit as participants equal numbers of persons with and without experience driving physical automobiles. During virtual driving, we expected that drivers and non-drivers would move differently, and that postural instabilities would precede the onset of motion sickness. Most importantly, we predicted that patterns of bodily movement would reveal statistically significant interactions between physical driving experience and motion sickness during virtual driving. That is, we predicted that the postural precursors of motion sickness during virtual driving would vary as a function of the presence or absence of decades of experience with physical driving.

We could not exercise experimental control over which participants became motion sick and which did not. Similarly, membership in the driver and non-driver groups could not be determined by random assignment. Yet, we were able to treat driving experience as an independent variable. Motion sickness status (well vs. sick) was a dependent variable in our analysis of the influence of driving experience on susceptibility to motion sickness in virtual vehicles. At the same time, motion sickness status was an independent variable in our analysis of relations between driving experience, postural activity, and motion sickness (for similar dual use of motion sickness as both a dependent variable and an independent variable, see [14,18,24,26]). For these reasons, ours was a quasi-experimental design.

## Materials and methods

### Participants

Twenty drivers (mean age = 50.83 ± 5.01 years; mean height = 163.81 ± 6.48 cm; mean weight = 62.43 ± 12.59 kg; 10 men and 10 women) and 20 non-drivers (mean age = 50.63 ± 4.69 years; mean height = 158.92 ± 6.38 cm; mean weight = 60.49 ± 10.06 kg; 10 men and 10 women) were recruited as participants. The mean age at which participants first obtained the driver’s license was 23.85 years (SD = 4.50 years). Each driver had held a current, valid driver’s license, on average, for 23.85 ± 4.50 years. Each driver reported that, over the preceding two months they had driven at least once each week and, on average, drove for 4.54 hours per week. Among the 20 non-drivers, 13 did not hold a driver’s license and had never driven any automobile, while the other 7 had held a valid driver’s license but reported that they had not driven for at least 15 years. All participants had normal or corrected-to-normal vision, and reported that they had no history of disease or malfunction of the vestibular apparatus, recurrent dizziness, or falls. Informed consent was obtained prior to participation. The sample size was identical to Chang et al. [12], and similar to other studies of motion sickness in virtual environments [14,24]. National Kaohsiung Normal University does not have an Institutional Review Board for behavioral science research. For this reason, the study protocol was approved by the Research Ethics Committee for Human Behavioral Sciences of National Cheng Kung University.

### Apparatus

The study was conducted using a standard Xbox system (Xbox 360 pro, Microsoft Corp), which included the game unit and a handheld device that participants used to control the game. The video and audio portions of the game were presented using a LED monitor (KDL-55NX720, Sony) that measured 139.67 cm diagonally (122 cm × 68 cm). Participants sat on a 46 cm high stool that did not support the torso. Participants rested their feet on the floor and were asked not to change their foot position during the session. The stool had four feet; the front two feet were placed on the line on the floor 105 cm away from the monitor. The visual angle of the screen was approximately 60° horizontal by 36° vertical.

Data on head and torso movement were collected using a magnetic tracking system (Flock of Birds, Ascension Technologies, Inc., Burlington, VT). One receiver was attached to a bicycle helmet worn by the participant, and another to the skin at the level of the 7^th^ cervical vertebra using cloth medical tape. The transmitter was located behind the participant’s head. Six degree-of-freedom position data were collected from each receiver at 60 Hz and stored for later analysis.

### Procedure

We separately assessed the incidence of motion sickness and the severity of symptoms. We assessed the incidence of motion sickness using a forced-choice, yes/no question, Are you motion sick? Participants who answered *yes* were assigned to sick group; all others were assigned to the well group. We assessed symptom severity using a modified version of the Simulator Sickness Questionnaire, or *SSQ* [27], which includes 16 items with a 4-point scale. The modification consisted of the inclusion of our forced-choice question about motion sickness incidence. The questions were translated into Chinese.

Incidence and severity were assessed more than once. After completing the informed consent procedure, participants filled out the SSQ (SSQ-1). The pre-exposure administration ensured that participants were not already motion sick, that they were familiar with the subjective symptoms of motion sickness, and also provided a baseline for comparison with post-exposure scores.

**Fig 1:**
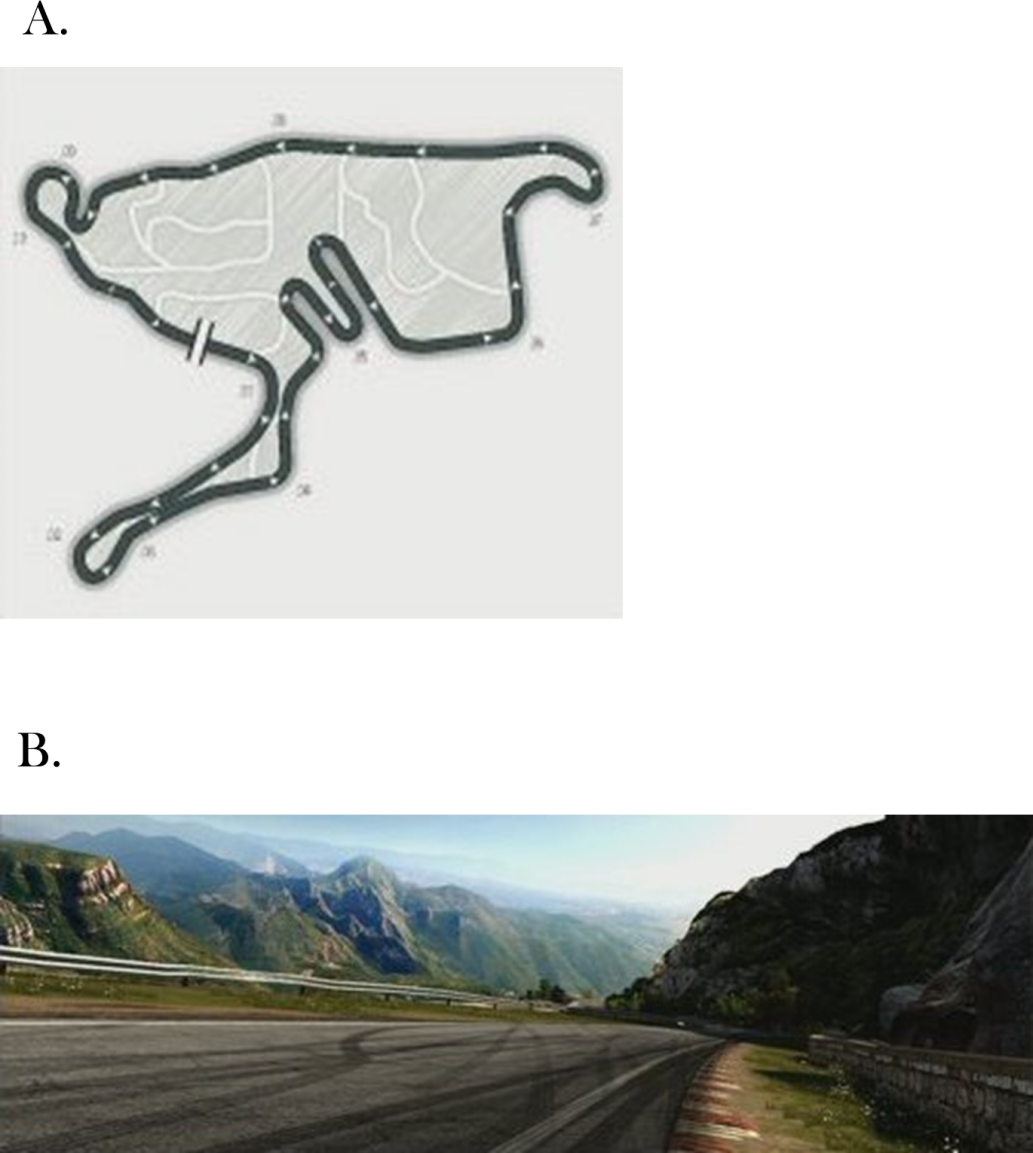
The driving game. (A) Overhead representation of the course (circuit). (B) Momentary driver’s-eye view.

Next, participants were given a brief introduction to the Xbox system and to the game and were then permitted to explore the game until they felt that they understood the rules and the use of the game pad. Participants played *Forza Motosport 3*, an auto racing game, using the game pad. Participants freely controlled both speed and steering. Positive acceleration (speeding up) was achieved with a button controlled by the right index finger, while negative acceleration (braking) was achieved via a button controlled by the left index finger. The left thumb operated a directional button that was used to control the right or left direction of the car. Participants drove the *Ford/ R3 714* over a 6.95 km *Extreme Circuit* in the *Camino Viejo de Montserrant* (Fig 1). The course traversed mountainous terrain, requiring frequent acceleration and braking. The camera/viewpoint was set at the driver’s seat, a first-person perspective. Participants were instructed to complete the designated course as quickly as possible. Participants played the game continuously for up to 40 min, restarting the game if necessary (i.e., if the game ended [12]). Data on head and torso movement were collected continuously from the beginning of to the end of game play (i.e., until the end of the 40-min session, or until the subject discontinued participation, whichever came first).

Once the game ended, participants’ game performance, including time elapsed in the present lap, the number of laps completed, and their fastest lap, was shown on the screen. At the end of 40 min (or at the time of discontinuation, whichever came first) participants completed the post-exposure SSQ (SSQ-2). Participants who stated that they were not motion sick at SSQ-2 were given a printed copy of the form (SSQ-3) and asked to fill it out immediately if they became motion sick after leaving the laboratory or, if they they did not become sick, to fill it out after 24 hours.

Before beginning game play, participants were told that they should stop playing immediately if they felt any symptoms of motion sickness, however mild. For this reason, all movement data included in our analyses were precursors to subjective symptoms of motion sickness.

### Data analysis

#### Subjective reports, discontinuation, and game performance

We included all participants in our analyses of motion sickness incidence, discontinuation, and symptom severity. We used χ^2^ statistics to analyze the data on motion sickness incidence. Data on symptom severity were evaluated using the Mann-Whitney test and the Wilcoxon signed ranks test. In evaluating SSQ data, we used the Total Severity Score, which was computed in the recommended manner [27]. We evaluated game performance in two ways. First, we computed the percent of participants who completed at least one lap. Second, we recorded the duration of fastest lap. We used χ^2^ statistics and the Mann-Whitney test to analyze these performance data.

#### Movement data

We separately evaluated the spatial magnitude of movement and its temporal dynamics. We evaluated the spatial magnitude of movement in terms of the standard deviation of position of the head and torso. We evaluated the temporal dynamics of movement in terms of α, the scaling exponent of value of detrended fluctuation analysis (DFA). DFA describes the relationship between the magnitude of fluctuations in postural motion and the time scale over which those fluctuations are measured [28]. The scaling exponent of DFA, α, is an index of long-range autocorrelation in the data, that is, the extent to which the data are self-similar (e.g., more periodic, or more predictable) over time. DFA has been widely used to evaluate the temporal dynamics of human movement in terms of standing body sway [29], and in relation to visually induced motion sickness [17,19–20]. For both spatial magnitude and temporal dynamics, we conducted separate analyses of variance (ANOVAs) for movement in the anteroposterior (AP) and mediolateral (ML) axes of the head and torso.

In our ANOVAs, we estimated the effect size using the partial η^2^ statistic. According to Cohen [30], values of partial η^2^ > 0.14 indicate a large effect, and values of partial η^2^ > .06 indicate a medium effect. When the sphericity assumption was violated, we used the Huynh-Feldt method [31]. The Huynh-Feldt method yields fractional degrees of freedom, which we report where appropriate.

## Results

### Motion sickness incidence and discontinuation

Participants who stated that they were motion sick at SSQ-2 or SSQ-3 were assigned to the Sick group. All others were assigned to the Well group. Prior to virtual driving, each subject stated that they were not motion sick. After virtual driving, the overall incidence of motion sickness was 52.5% (21/40). This incidence did not differ from that observed among young adult participants (62.5%; [12]), *χ*^2^ (1) = 0.82, *p* = .37. Thirteen drivers (65%) stated that they were motion sick, including 5 males and 8 females. Eight non-drivers (40%) stated that they were motion sick, including 2 males and 6 females. Each participant who stated that they were motion sick did so at SSQ-2. No participant reported motion sickness at SSQ-3. Using a 2 × 2 contingency table, the incidence of motion sickness did not differ between drivers and non-drivers, *χ*^2^ (1) = 2.51, *p* = .11. However, motion sickness incidence was greater among women (70%) than among men (35%), *χ*^2^ (1) = 4.91, *p* = .027.

For sick drivers, the mean time of discontinuation was 13.72 ± 6.56 min. Each driver who stated that they were well completed the game session. Eleven of 12 well non-drivers completed the game session. One well non-driver discontinued (at 38.70 min), without giving a reason. Each sick non-driver discontinued, with a mean time of discontinuation of 24.06 ± 8.77 minutes. Among participants in the Sick group, mean exposure time was greater for non-drivers than for drivers, *t* (19) = -3.090, *p* = .006.

### Game performance

The percentage of participants who completed at least one lap did not differ between sick drivers (8/13, 61.54%) and well drivers (7/7, 100%), *χ*^2^ (1) = 3.59, *p* > .05. However, this percentage was significantly lower for sick non-drivers (5/8, 62.5%) than for well non-drivers (7/7, 100%), *χ*^2^ (1) = 5.29, *p* = .02.

Among those who finished at least one lap, the fastest lap in minutes did not differ between sick drivers (7.88 ± 3.13 min.) and well drivers (12.29 ± 6.98 min.), *U* = 17.00, or between sick non-drivers (10.41 ± 3.06 min.) and well non-drivers (11.88 ± 4.76 min.), *U* = 21.00, *p* > .05. For the sick group, the fastest lap in minutes did not differ between drivers and non-drivers (mean drivers = 7.88 ± 3.13 min, mean non-drivers = 10.41 ± 3.06 min), *U* = 10.00. This was true also for the well group (mean drivers = 12.29 ± 6.98 min, mean non-drivers = 11.88 ± 4.76 min), *U* = 37.00, *p* > .05.

### Symptom severity

**Fig 2:**
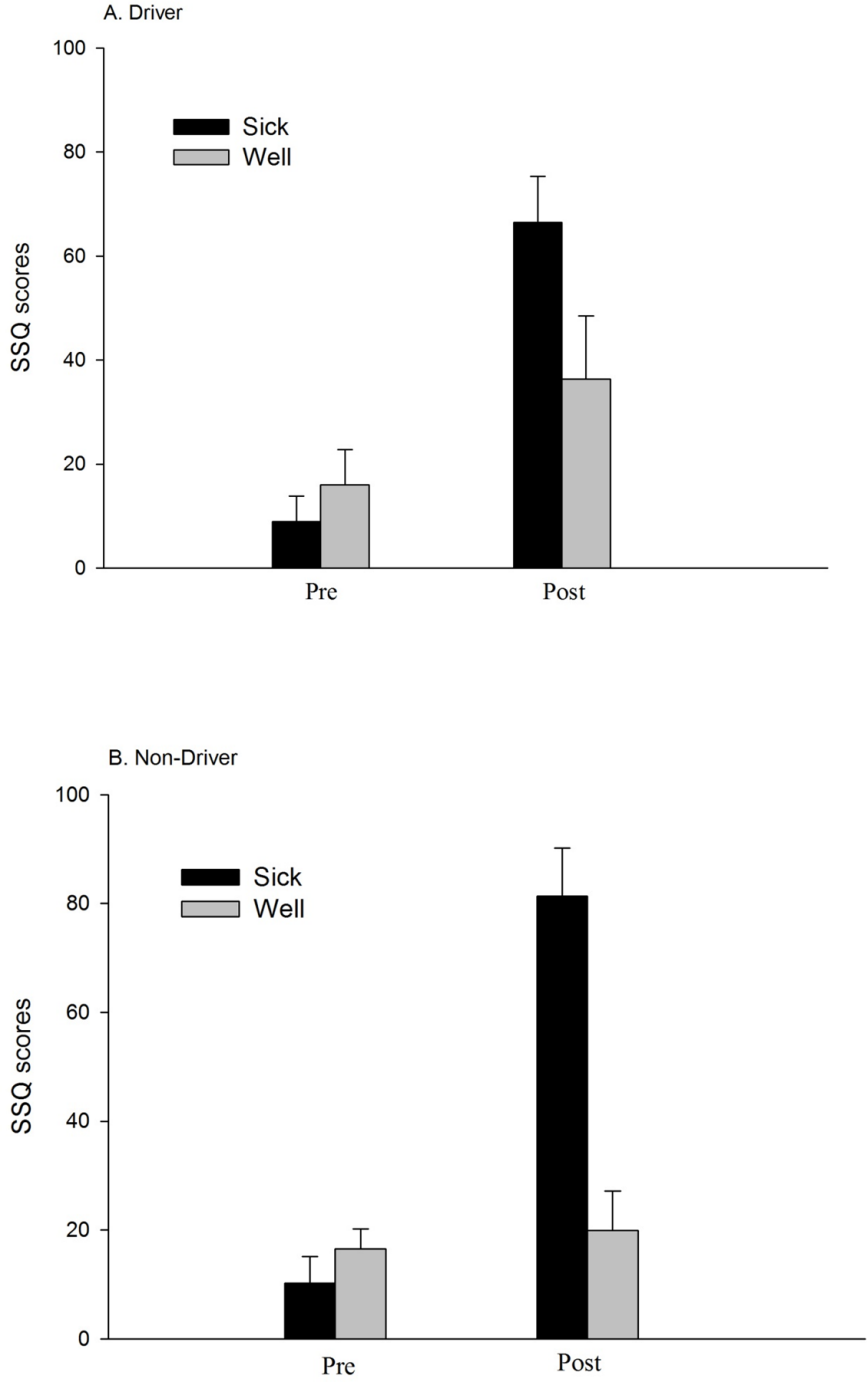
Symptom severity (SSQ Total Severity Scores) for the well and sick groups. (A) Drivers. (B) Non-Drivers. Pre: Pre-exposure. Post: Post-exposure.

Data on symptom severity are summarized in Fig 2. As in Chang et al. [12], pre-exposure SSQ scores did not differ between the sick and well groups for drivers, *U* = 36.50, *p* > .05, or for non-drivers, *U* = 37.00, *p* > .05.

Among sick drivers, post-exposure scores were higher than pre-exposure scores, *Z* = -3.18, *p* = .001, consistent with Chang et al. [12]. Among well drivers, post-exposure scores were higher than pre-exposure scores, *Z* = -2.20, *p* = .028. Among drivers, post-exposure SSQ scores did not differ between the sick and well groups, *U* = 21.5, *p* > .05 (*p* = .056). These latter two results differed from Chang et al. [12]. Taken together, these results indicate that among drivers in the present study (i.e., for middle-aged adults), the incidence of motion sickness and the severity of symptoms were independent.

Among sick non-drivers, post-exposure scores were higher than pre-exposure scores, *Z* = -2.52, *p* = .012, consistent with Chang et al. [12]. Among well non-drivers, pre-exposure SSQ scores did not differ from post-exposure SSQ scores, *Z* = -0.46, *p* >.05, unlike Chang et al. [12]. Among non-drivers, post-exposure SSQ scores were greater for the sick group than for the well group, *U* = 2.50, *p* < .001, consistent with Chang et al. Taken together, these results indicate that among middle-aged non-drivers the incidence of motion sickness was associated with an increase in symptom severity.

Post-exposure SSQ scores did not differ between sick drivers and sick non-drivers, *U* = 39.50, *p* > .05. Post-exposure SSQ scores did not differ between well drivers and well non-drivers, *U* = 28.00, *p* > .05. Collapsed across sickness groups, post-exposure SSQ scores did not differ between drivers and non-drivers, *U* = 150.50, *p* > .05. Each of these results is consistent with Chang et al. [12]. These results indicate that the severity of post-exposure symptoms did not differ between drivers and non-drivers.

We conducted two comparisons between the present study and that of Chang et al. [12], that is, between young and middle-aged adults. Among participants in the sick group, post-exposure SSQ scores did not differ between young and middle-aged drivers, *U* = 69.5, p > .05 (*p* = .448). Similarly, among participants in the sick group, post-exposure SSQ scores did not differ between young and middle-aged non-drivers, *U* = 38.5, p > .05 (*p* = .473).

### Movement data

One driver reported motion sickness after less than 6 minutes of virtual driving and, for this reason, was excluded from movement analysis. For the remaining 12 sick drivers, the mean time of discontinuation was 14.44 ± 6.29. As in Chang et al. [12], we defined windows for the well groups based on the mean exposure time of sick drivers. Accordingly, Window 1 comprised the first 120 seconds of game play. Window 2 ran from 6.22 to 8.22 minutes, and Window 3 ran from 12.44 minutes to 14.44 minutes.

#### Positional variability

**Fig 3:**
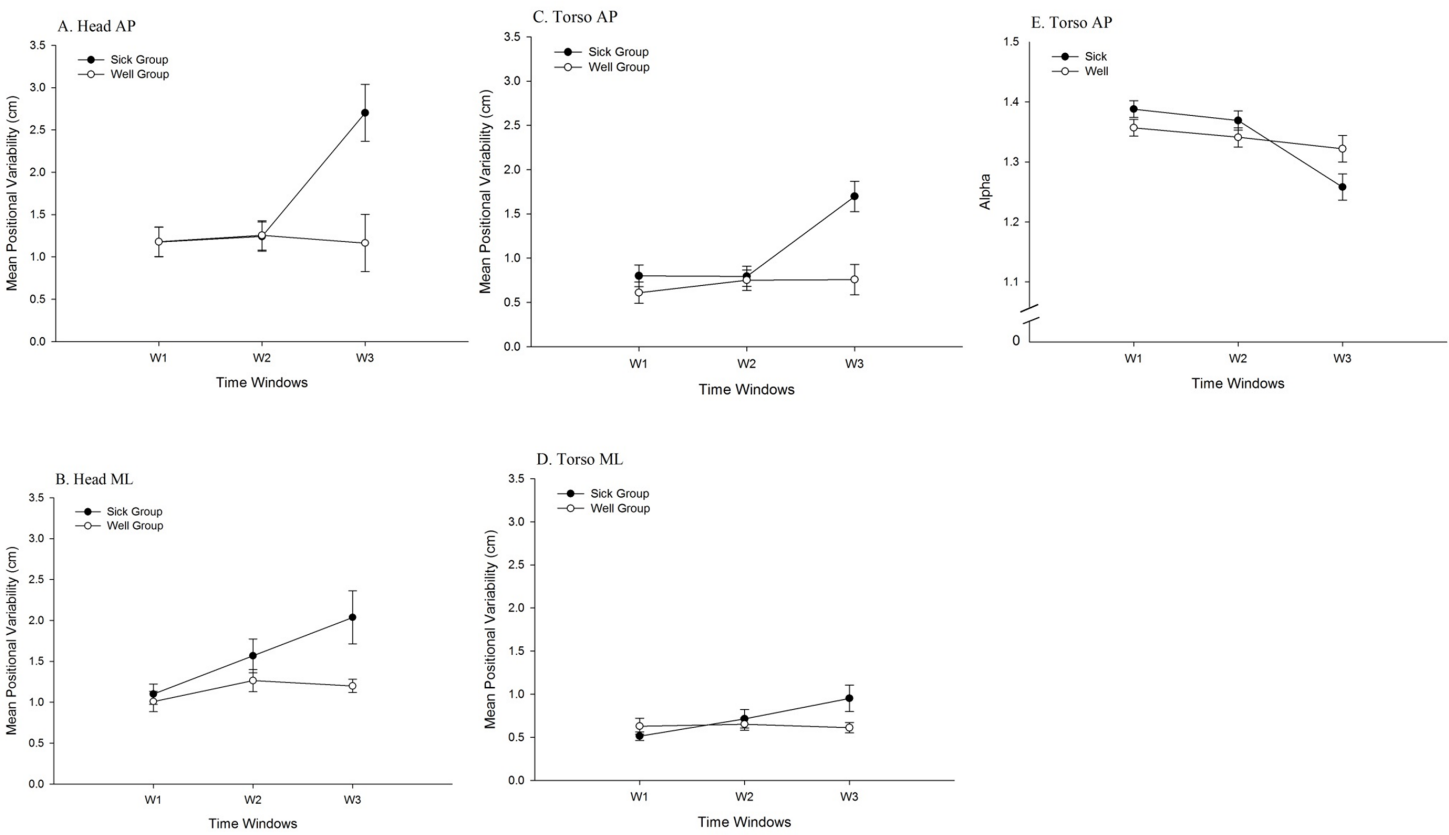
Movement data, illustrating the statistically significant 2-way interactions between Time Windows and Sickness Groups. (A) Positional variability of the head in the AP axis. (B) Positional variability of the head in the ML axis. (C) Positional variability of the torso in the AP axis. (D) Positional variability of the torso in the ML axis. (E) Temporal dynamics (α of DFA) of the torso in the AP axis.

**Fig 4:**
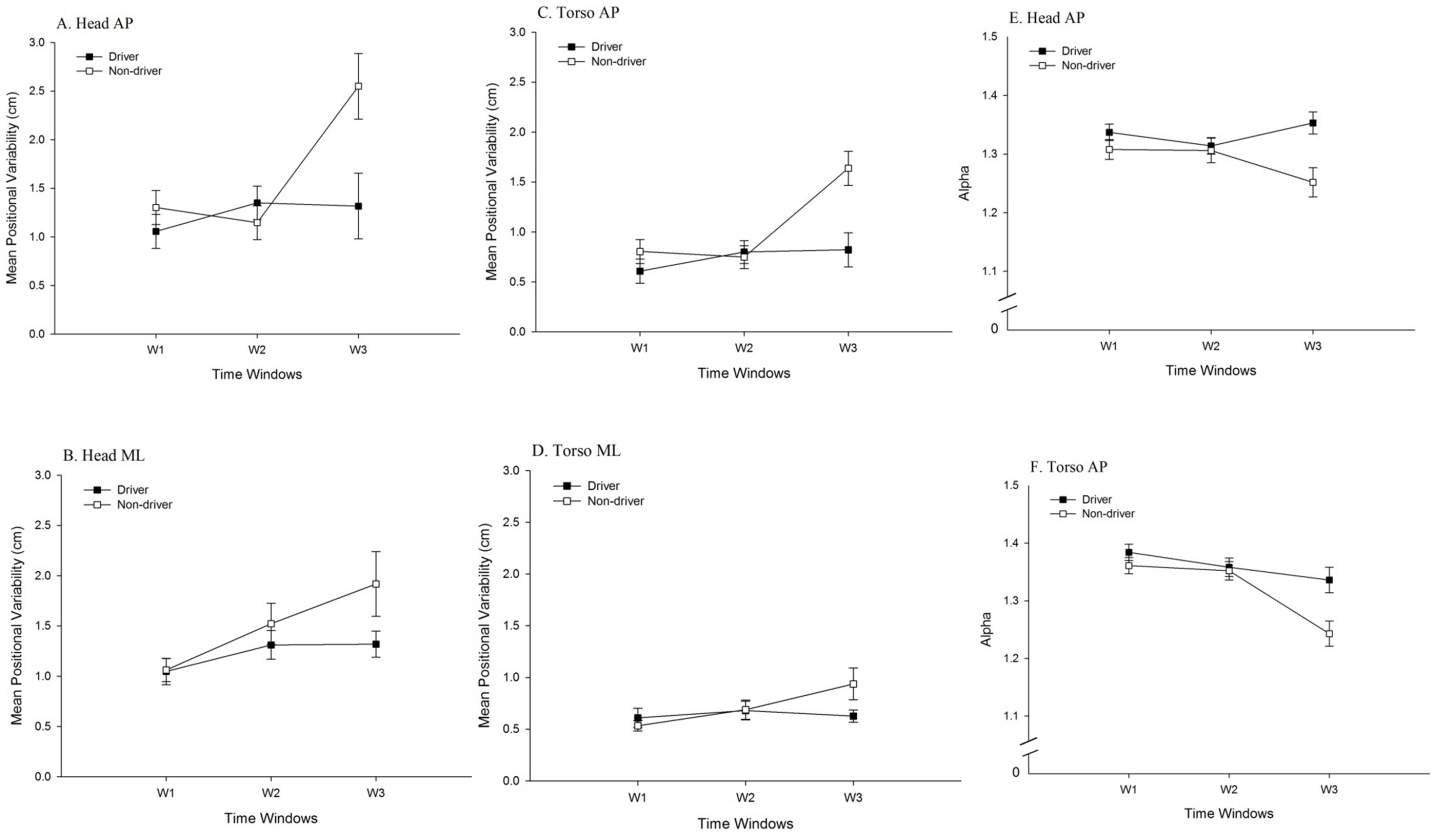
Movement data, illustrating the statistically significant 2-way interactions between driving experience and Time Windows. (A) Positional variability of the head in the AP axis. (B) Positional variability of the head in the ML axis. (C) Positional variability of the torso in the AP Axis. (D) Positional variability of the torso in the ML axis. (E) Temporal dynamics (α of DFA) of the head in the AP axis. (F) Temporal dynamics (α of DFA) of the torso in the AP axis.

**Fig 5:**
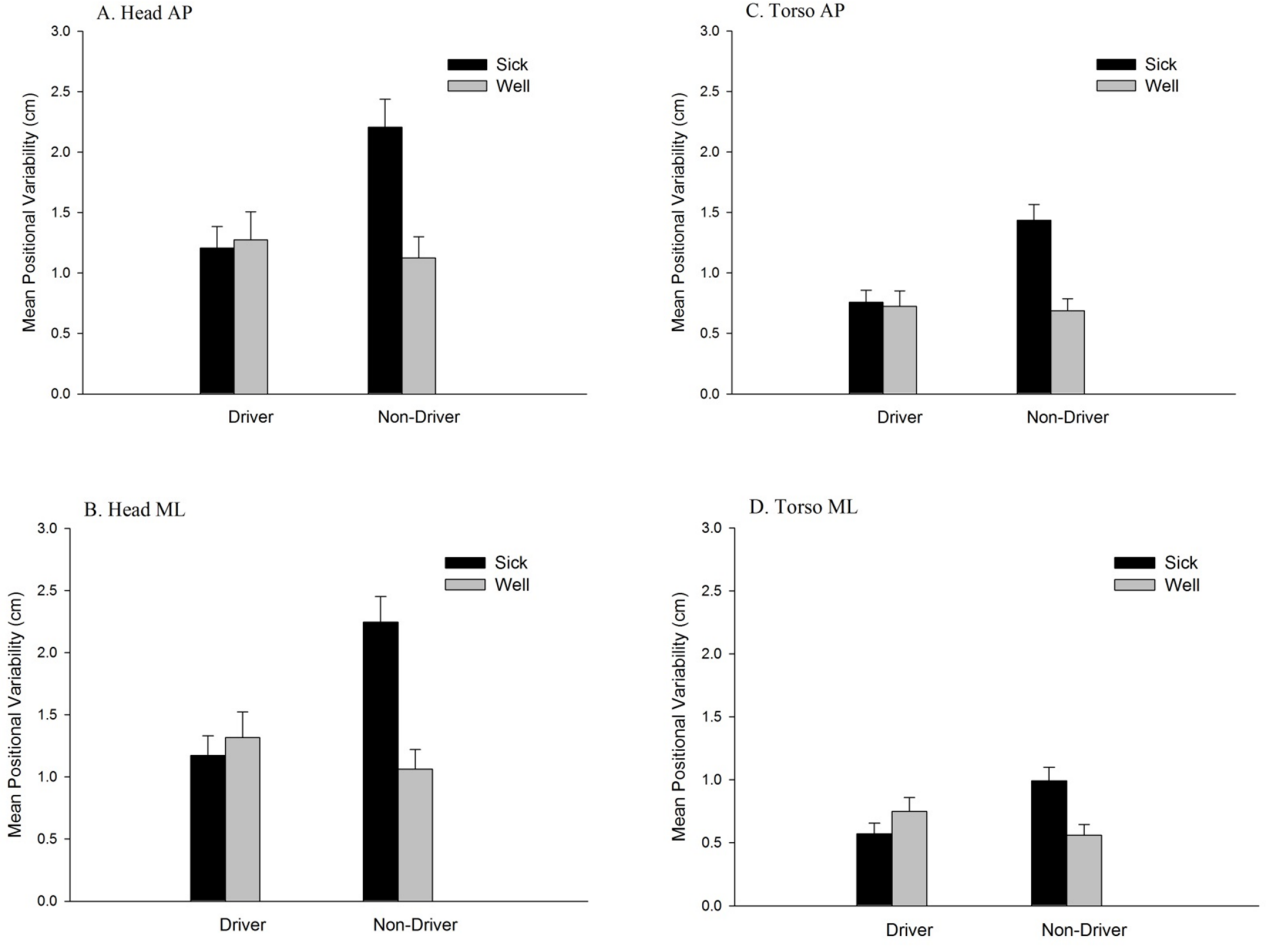
Positional variability, illustrating the statistically significant 2-way interactions between driving experience and Sickness Groups. (A) The head in the AP axis. (B) The head in the ML axis. (C) The torso in the AP axis. (D) The torso in the ML axis.

**Fig 6:**
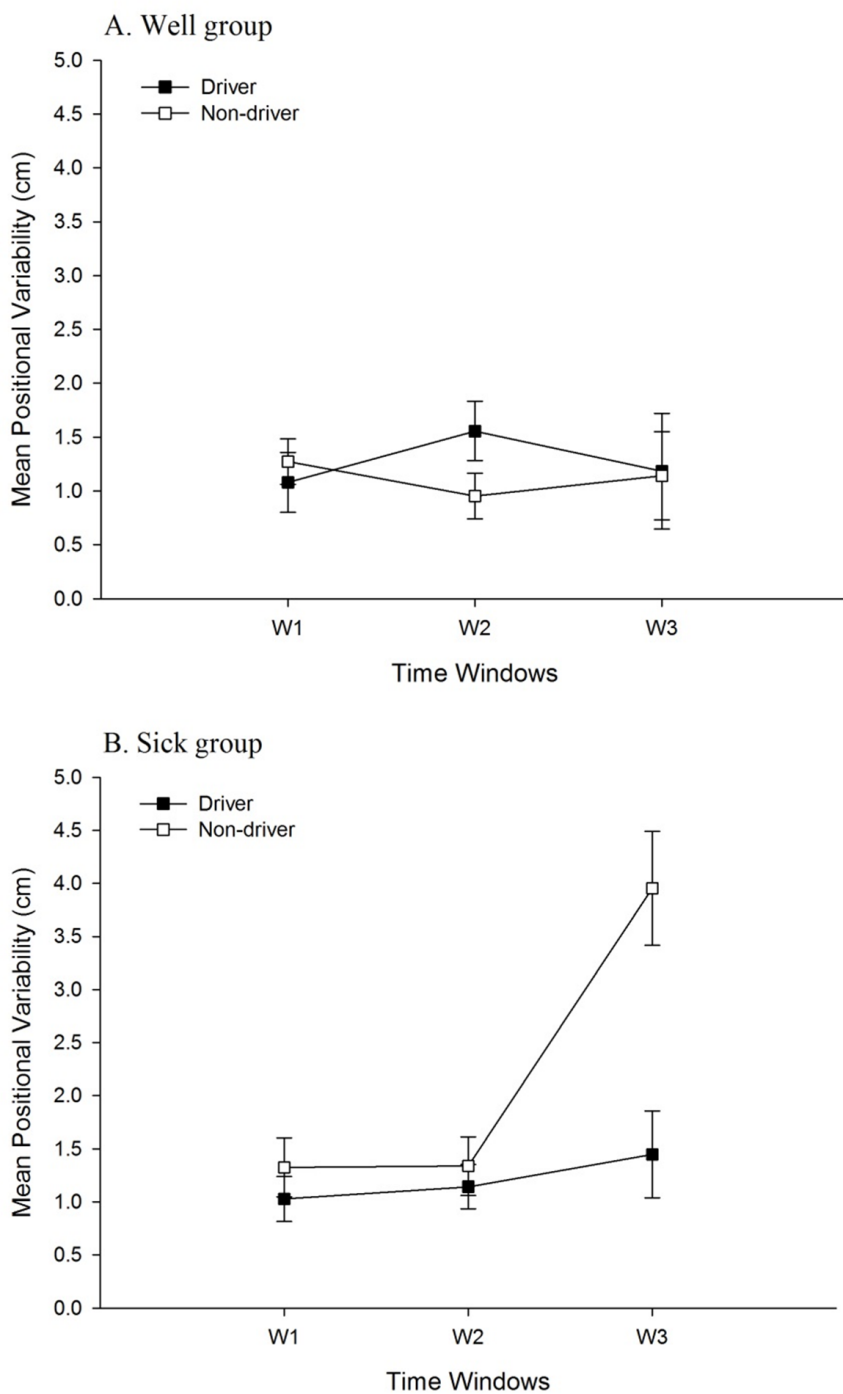
Positional variability of the head in the AP axis. The figure illustrates the statistically significant 3-way interaction between Driving Experience, Time Windows, and Sickness Groups. (A) Well group. (B) Sick group.

For head movement in the AP axis, the main effect of Sickness Groups was significant, *F* (1, 34) = 6.08, *p* = .019, partial η^2^ = .15. Positional variability in the Sick group (1.58 ± 1.49 cm) was greater than in the Well group (1.18 ± 0.72 cm). The main effect of Driving Experience was significant, *F* (1, 34) = 4.26, *p* = .047, partial η^2^ = .11. Positional variability among non-drivers (1.52 ± 1.53 cm) was greater than among drivers (1.23 ± 0.66 cm). The main effect of Time Windows was significant, *F* (1.67, 56.78) = 6.27, *p* = .006, partial η^2^ = .16 (Table 1). The Sickness Groups × Time Windows interaction was significant, *F* (1.67, 56.78) = 7.18, *p* = .003, partial η^2^ = .17 (Fig 3A). The Time Windows × Driving Experience interaction was significant, *F* (1.67, 56.78) = 4.88, *p* = .015, partial η^2^ = .13 (Fig 4A). In addition, the Sickness Groups × Driving Experience interaction was significant, *F*(1, 34) = 7.80, *p* = .009, partial η^2^ = .19 (Fig 5A). Finally, the Sickness Groups × Driving Experience × Time Windows interaction was significant, *F* (1.67, 56.78) = 3.59, *p* = .042, partial η^2^ = .10. As shown in Fig 6, at Window 3, positional variability was elevated among non-drivers in the Sick group.

**Table 1:** Statistically significant main effects of Time Windows on spatial and temporal measures of movement during virtual driving.

				W1	W2	W3	Post-hoc tests
Variable							
Positional variability (cm)							
	Head						
			AP	1.17 ± 0.72	1.20 ± 0.73	1.76 ± 1.73	W3 > W1 = W2
			ML	1.05 ± 0.53	1.42 ± 0.77	1.62 ± 1.10	W3 = W2 > W1
	Torso						
			AP	0.70 ± 0.50	0.75 ± 0.47	1.12 ± 0.96	W3 > W1 = W2
			ML	0.57 ± 0.33	0.68 ± 0.39	0.78 ± 0.53	W3 > W1
α of DFA							
	Head						
			ML	1.35 ± 0.05	1.25 ± 0.09	1.25 ± 0.09	W1 > W2 = W3
	Torso						
			AP	1.37 ± 0.06	1.35 ± 0.07	1.30 ± 0.11	W1 = W2 > W3

*Note*. W1 = Window 1; W2 = Window 2; W3 = Window 3; ± denotes standard deviation.

For head movement in the ML axis, the main effect of Sickness Groups was significant, *F* (1, 34) = 7.97, *p* = .008, partial η^2^ = 0.19; positional variability was greater for the Sick group (1.57 ± 1.07 cm) than for the Well group (1.16 ± 0.51 cm). The main effect of Driving Experience was significant, *F* (1, 34) = 5.00, *p* = .032, partial η^2^ = 0.13; positional variability was greater among Non-Drivers (1.50 ± 1.05 cm), than among Drivers (1.23 ± 0.59 cm). The main effect of Time Windows was significant, *F* (2, 68) = 13.05, *p* < .001, partial η^2^ = 0.28 (Table 1). The Sickness Groups × Time Windows interaction was significant, *F* (2, 68) = 8.18, *p* = .001, partial η^2^ = 0.19 (Fig 3B). In addition, the Driving Experience × Time Windows interaction was significant, *F* (2, 68) = 6.04, *p* = .004, partial η^2^ = 0.15 (Fig 4B). Finally, the Sickness Groups × Driving Experience interaction was significant, *F* (1, 34) = 13.02, *p* = .001, partial η^2^ = 0.28 (Fig 5B). Among non-drivers, the sick group showed larger positional variability than did the well group, while among drivers, the trend was reversed.

**Fig 7:**
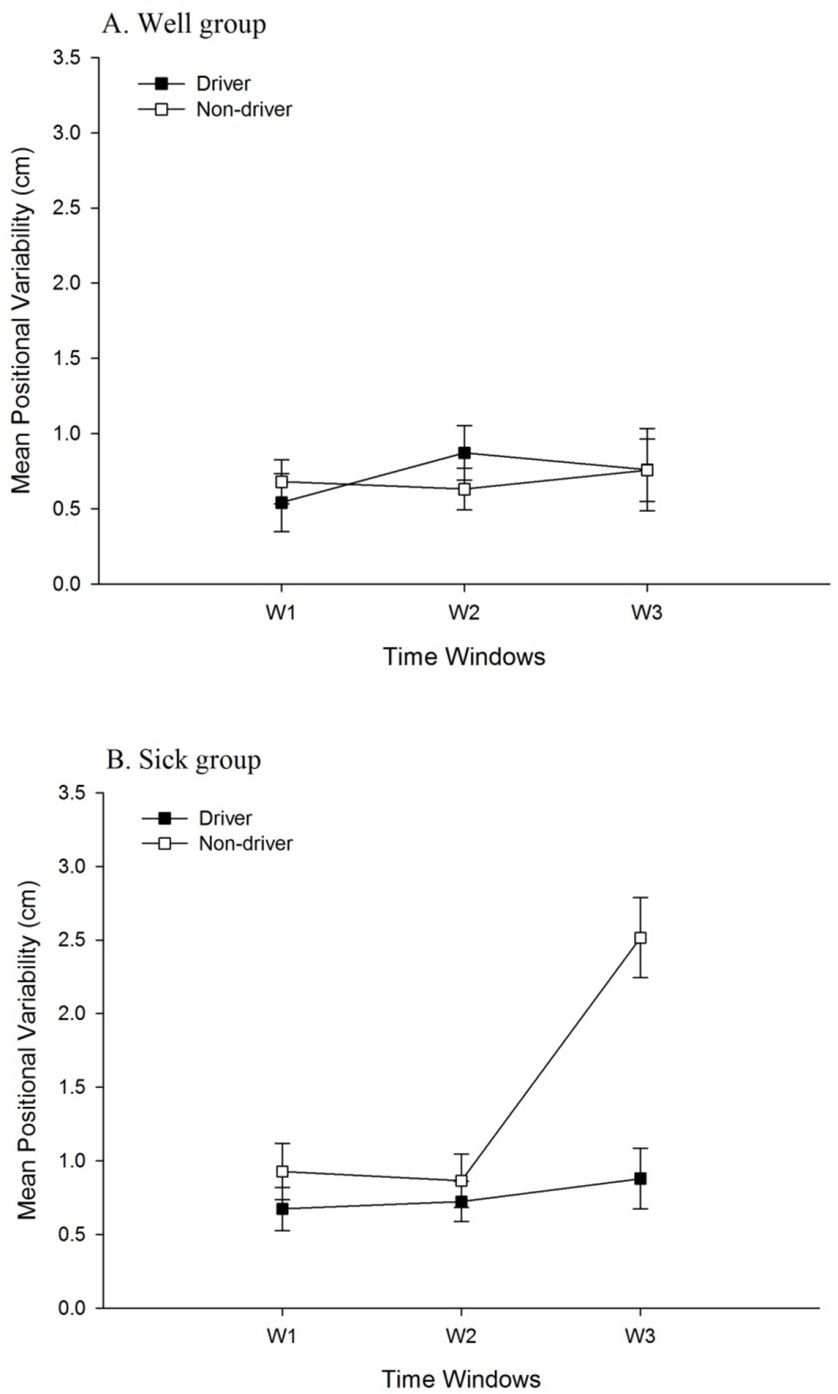
Positional variability of the torso in the AP axis. The figure illustrates the statistically significant 3-way interaction between Driving Experience, Time Windows, and Sickness Groups. (A) Well group. (B) Sick group.

For torso movement in the AP axis, the main effect of Sickness Groups was significant, *F* (1, 34) = 11.78, *p* = .002, partial η^2^ = 0.26; positional variability was greater for the Sick group (1.01 ± 0.88 cm) than for the Well group (0.70 ± 0.42 cm). The main effect of Driving Experience was significant, *F* (1, 34) = 7.90, *p* = .008, partial η^2^ = 0.19; positional variability was greater among Non-Drivers (0.96 ± 0.86 cm), than among Drivers (0.75 ± 0.49 cm). The main effect of Time Windows was significant, *F* (1.82, 61.98) = 8.70, *p* = .001, partial η^2^ = 0.20 (Table 1). The Sickness Groups × Time Windows interaction was significant, *F* (1.82, 61.98) = 6.20, *p* = .005, partial η^2^ = 0.15 (Fig 3C). The Time Windows × Driving Experience interaction was significant, *F* (1.82, 61.98) = 5.35, *p* = .009, partial η^2^ = 0.14 (Fig 4C). In addition, the Sickness Groups × Driving Experience interaction was significant, *F* (1, 34) = 9.74, *p* = .004, partial η^2^ = 0.22 (Fig 5C). Finally, the Sickness Groups × Driving Experience × Time Windows interaction was significant, *F* (1.82, 61.98) = 4.45, *p* = .018, partial η^2^ = 0.12 (Fig 7).

For torso movement in the ML axis, the main effect of Time Windows was significant, *F* (2, 68) = 5.97, *p* = .004, partial η^2^ = 0.15 (Table 1). The Sickness Groups × Time Windows interaction was significant, *F* (2, 68) = 10.10, *p* < .001, partial η^2^ = 0.23 (Fig 3D). The sick individuals had greater positional variability at Window 3 as compared to Window 1. At Window 3, the sick individuals showed greater positional variability as compared to the well ones. In addition, the Driving Experience × Time Windows interaction was significant, *F* (2, 68) = 8.74, *p* < .001, partial η^2^ = 0.21 (Fig 4D). Finally, the Sickness Groups × Driving Experience interaction was significant, *F* (1, 34) = 9.74, *p* = .004, partial η^2^ = 0.22 (Fig 5D). Among non-drivers, participants in the sick group exhibited greater positional variability than did participants in the well group, while the trend was reversed for the drivers.

#### Temporal dynamics

For head movement in the AP axis, the main effect of Driving Experience was significant, *F* (1, 34) = 6.75, *p* = .014, partial η^2^ = 0.17. The α parameter was greater among drivers (1.33 ± 0.07 cm) than among non-drivers (1.29 ± 0.10 cm), indicating greater self-similarity in the movement of drivers than in the movement of non-drivers. In addition, the Driving Experience × Time Windows interaction was significant, *F* (1.71, 58.06) = 7.00, *p* = .003, partial η^2^ = .17 (Fig 4E).

For head movement in the ML axis, the main effect of Time Windows was significant, *F* (2, 68) = 19.15, *p* < .001, partial η^2^ = 0.36 (Table 1).

**Fig 8:**
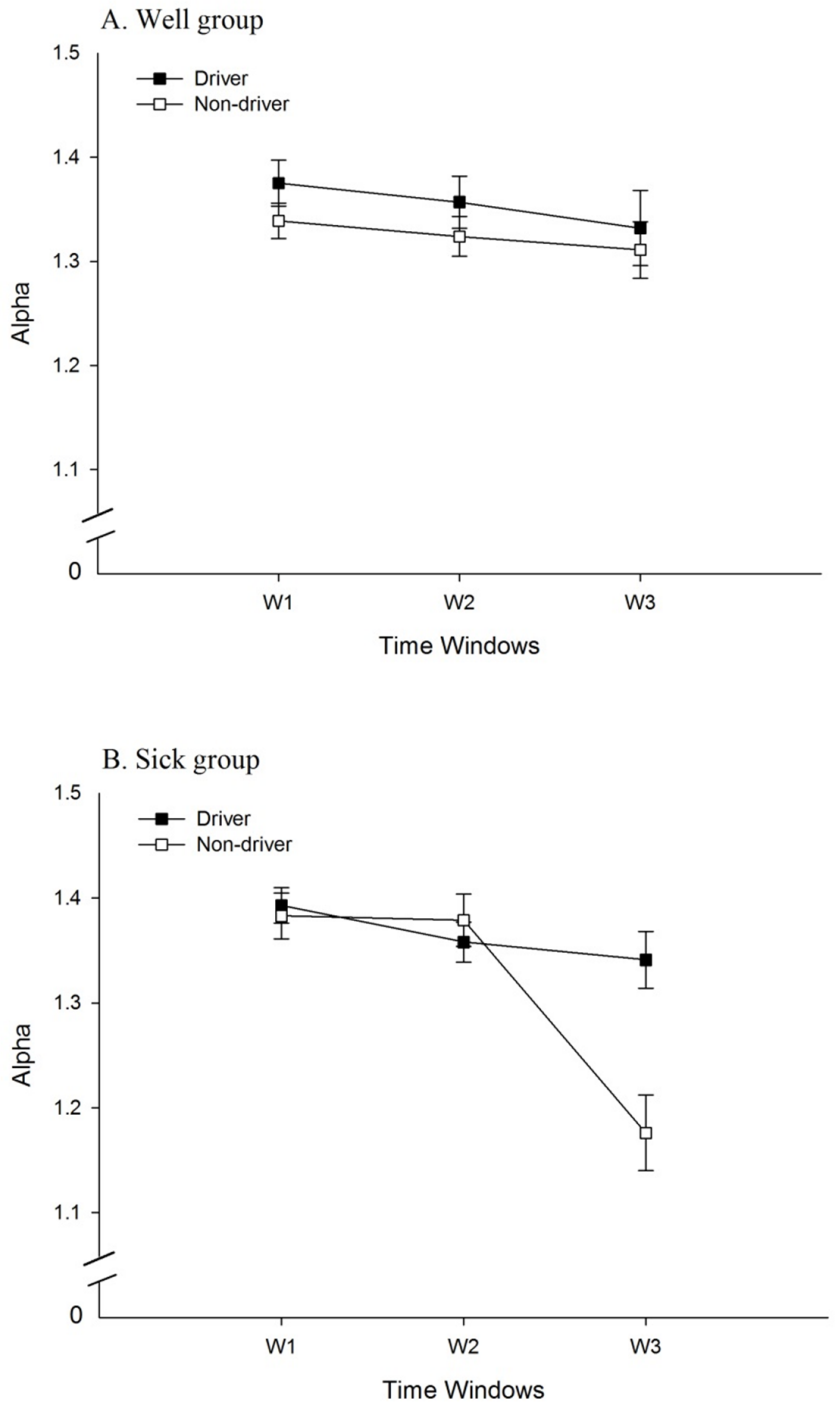
Temporal dynamics (α of DFA) of the torso in the AP axis. The figure illustrates the statistically significant 3-way interaction between Driving Experience, Time Windows, and Sickness Groups. (A) Well group. (B) Sick group.

For torso movement in the AP axis, the main effect of Driving Experience was significant, *F* (1, 34) = 5.33, *p* = .027, partial η^2^ = 0.14, with the α parameter being greater for drivers (1.36 ± 0.08 cm) than for non-drivers (1.32 ± 0.09 cm). The main effect of Time Windows was significant, *F* (1.76, 59.80) = 16.06, *p* < .001, partial η^2^ = 0.32 (Table 1). The Sickness Groups × Time Windows interaction was significant, *F* (1.76, 59.80) = 6.10, *p* = .005, partial η^2^ = 0.15 (Fig 3E). In addition, the Driving Experience × Time Windows interaction was significant, *F* (1.76, 59.80) = 4.47, *p* = .019, partial η^2^ = 0.12 (Fig 4F). Finally, the Time Windows × Sickness Groups × Driving Experience interaction was significant, *F* (1.76, 59.80) = 6.15, *p* = .005, partial η^2^ = 0.15 (Fig 8).

There were no significant effects for torso movement in the ML axis.

## Discussion

Middle-aged adults drove a virtual automobile in a driving video game. Drivers had approximately 30 years of experience driving physical automobiles, while non-drivers had not driven a physical automobile for at least 15 years. During virtual driving, the incidence of motion sickness and the severity of motion sickness symptoms did not differ between drivers and non-drivers, but drivers who reported motion sickness discontinued participation earlier than sick non-drivers. The kinematics of the head and torso during game play were strongly influenced by our manipulations, including differences between Well and Sick participants, and differences between drivers and non-drivers. Of special relevance, we found several statistically significant interactions between these two variables. We discuss these results in turn.

### Incidence, severity, discontinuation, and game performance

The incidence of motion sickness did not differ between drivers and non-drivers, and the severity of symptoms did not differ between sick drivers and sick non-drivers. These results replicated Chang et al. [12], and extend their findings to middle-aged adults with three decades of physical driving experience. In terms of motion sickness, we found only one statistically significant effect related to physical driving experience: In the Sick group, drivers discontinued earlier than non-drivers. This effect was novel and indicates that several decades of physical driving experience was not entirely irrelevant to motion sickness during virtual driving. Separately, we replicated the common finding that women are more susceptible than men [11,22].

Participants in the Sick group were less likely to finish at least one lap than participants in the Well group, but this was true only for non-drivers. Accordingly, this result reveals an effect of driving experience on performance in the virtual driving game.

Some studies have reported that motion sickness in driving simulators is more common among older adults [6]. However, the empirical evidence for this effect is weak. Brooks, Tyrrell, and Stephens [32] used an indirect measure of motion sickness incidence; the number of participants who terminated their participation before the end of the protocol. Using this measure, the incidence of motion sickness was 7% for younger adults (mean age = 19.3 years), and 17% for middle-aged adults (mean age = 43.2 years). Based on the data provided by Brooks et al., we compared these rates and found that they did not differ, χ ^2^ (1) = 0.653, *p* = .419. In the present study, the incidence of motion sickness among middle-aged participants during virtual driving did not differ from that reported by Chang et al. [12], using the same driving game and study protocol with young adults. Accordingly, with respect to the incidence of motion sickness, our results are compatible with those of Brooks et al. [32].

### Movement during virtual driving

#### Main effects of time windows

Movement evolved over time during virtual driving, as reflected by several main effects of Time Windows (Table 1). In general, Table 1 shows that the spatial magnitude of movement tended to increase over time, while the self-similarity of movement tended to decrease over time. These effects replicate numerous studies [19,24,33], and confirm that some time-related changes in movement are independent of motion sickness status (well vs. sick). With young adult participants, Chang et al. [12] found no main effects of time windows; that is, for their young adult participants, movement did not evolve over time as a simple function of exposure to virtual driving. Taken together, the two studies suggest that chronological age altered the evolution of participants’ movement patterns over the course of time during virtual driving.

#### Effects of driving experience

Main effects of driving experience were significant in the positional variability of the head, in both the AP and ML axes, and of the torso in the AP axis. In each of these cases, positional variability was greater among non-drivers than among drivers. That is, approximately 30 years of experience driving physical automobiles led to a reduction in the spatial magnitude of body movement during virtual driving. In addition, the main effect of driving experience was significant for the temporal dynamics of the head and torso in the AP axis; in each case, the α parameter revealed that the self-similarity of movement was greater among drivers than among non-drivers. We also found that the evolution of movement over differed between drivers and non-drivers, as revealed by statistically significant Driving Experience × Time Windows interactions for both positional variability and temporal dynamics (Fig 4). These effects are novel, and indicate that decades of physical driving experience altered body movement during virtual driving, independent of motion sickness incidence.

#### Effects relating to motion sickness

We found statistically significant main effects of sickness groups for positional variability of the head in both the AP and ML axes, and of the torso in the AP axis. In each case, positional variability was greater among participants who later became sick than those who did not. As main effects, these postural precursors of motion sickness replicate many previous studies [17–19,21–23,33]. As shown in Fig 3, significant Sickness Groups × Time Windows interactions were found in both positional variability (head AP and ML, torso AP and ML) and in temporal dynamics (torso movement in the AP axis). These effects also replicated previous studies [14,20,24,33], showing that changes in movement over time differed between the Well and Sick groups.

#### Interactions between motion sickness incidence and driving experience

Of greatest relevance to our hypotheses in this study, physical driving experience influenced the patterns of body movement that preceded motion sickness, in statistically significant interactions. Some effects of this kind were reported by Chang et al. [12], but for middle-aged adults the present effects are novel. With one exception (Fig 5C), for 2-way interactions the differences in movement between well and sick drivers and non-drivers were qualitative. In addition, statistically significant 3-way interactions revealed that relations between driving experience and motion sickness status evolved over time during virtual driving (Figs 6–8). Two of these interactions were in positional variability (Figs 6 and 7), while one occurred in temporal dynamics (Fig 8). The number of these statistically significant interactions exceeds those found for young adults [12]. In addition, the statistically significant 3-way interactions were not observed in the earlier study, and are entirely novel. Taken together, these effects demonstrate that among middle-aged adults physical driving experience was related to very substantial differences in both the spatial magnitude and temporal dynamics of body movement between participants who became motion sick and those who did not, when driving a virtual automobile.

### Theoretical implications

It is well known that, for persons riding in the same vehicle, those in control of the vehicle, such as drivers, are less likely to experience motion sickness than those not in control, such as passengers [13]. Recent research has demonstrated that these control-related variations in susceptibility are related to (and preceded by) patterns of body movement that differ between drivers and passengers [14,24,34]. In the current study, we documented a related effect; that postural precursors of motion sickness while driving a virtual automobile are powerfully (i.e., qualitatively) influenced by participants’ prior experience controlling physical automobiles. Our results suggest that these effects may be related to the experience of driving over very long time (decades). Overall, the results are consistent with the postural instability theory of motion sickness, and offer little support for theories based on the concept of intersensory conflict.

As in Chang et al. [12], the results of the present study challenge the parsimony of theories of motion sickness etiology that rely on the concept of intersensory conflict [3–4]. The movement effects that we observed occurred despite the fact that motion sickness incidence did not differ between drivers and non-drivers. We found evidence that driving experience effected the duration of exposure required to generate motion sickness, but this effect provides (at best) weak support for the intersensory conflict theory.

Any expectations about intersensory stimulation that existed in our participants must have differed between drivers and non-drivers, due to the fact that drivers had decades of experience driving physical automobiles, while non-drivers had not driven for at least 15 years and, in many cases, had never driven. Our results revealed no difference in susceptibility to motion sickness, during virtual driving, between drivers and non-drivers. By stark contrast, we found many differences between drivers and non-drivers in terms of movement patterns during virtual driving. Of greatest theoretical importance were statistically significant interactions between physical driving experience and motion sickness incidence during virtual driving. These interactions make it clear that decades of physical driving created differences between drivers and non-drivers in the quantitative kinematics of postural control during virtual driving. Given these effects in postural activity, the absence of any difference in motion sickness incidence suggests that hypothetical internal expectations about intersensory relations (alleged to cause motion sickness) appear to have had no effect upon the control of movement. In other words, hypothetical internal expectations about patterns of intersensory stimulation appear to have acted exclusively to yield motion sickness, and to have been unrelated to control of the body in interacting with the virtual environment. This pattern of results is consistent with an argument offered by Stoffregen and Riccio ([15], p. 183–184) that the intersensory conflict theory is unparsimonious, in this case because the hypothetical expectations that are alleged to give rise to intersensory conflict (and, ultimately, to motion sickness) appear to have no other function. The results of the present study provide perhaps the strongest and most direct evidence for this lack of parsimony in the intersensory conflict theory.

It is important to emphasize that all of our participants had extensive experience traveling in physical automobiles. Thus, the differences in movement that we observed between drivers and non-drivers cannot be attributed to “experience in automobiles”, but must be attributed to experience *controlling* automobiles. Our results show, for middle-aged adults, that the control of physical automobiles affected the quantitative kinematics of the body during the control of a virtual automobile.

In the present study, as in Chang et al. [12], all participants drove a virtual automobile. In future research, it will be interesting to examine the converse situation, that is, to expose persons with and without experience driving physical automobiles to a virtual automobile as passengers, rather than as drivers (cf. [13–14]).

## Conclusion

It is widely believed that experience controlling physical vehicles influences the risk of motion sickness when controlling a virtual vehicle. However, in most previous research, control experience has been confounded with age [6,10]. In the context of virtual driving, we separated middle-aged participants’ experience driving physical automobiles from their chronological age. We found no evidence that several decades of experience of driving a physical automobile influenced the incidence or severity of motion sickness while driving a virtual automobile. The principal limitation of our study was that we did not include a group of older adults (e.g., over 65 years of age). In future studies, it will be important to compare persons in this age group with and without driving experience. The incidence of motion sickness among our middle-aged participants did not differ from that reported by Chang et al. [12] for young adults, which suggests that any increase in motion sickness among persons over 65 years of age will likely be related to chronological age, rather than to driving experience.

We found only modest evidence that experience driving physical automobiles influenced the actual occurrence of motion sickness: Individuals with driving experience who became motion sick did so more rapidly (i.e., discontinued sooner) than individuals who became sick but who had never driving an automobile, but there were no differences in terms of the overall incidence of motion sickness, or the severity of symptoms.

We did not compare participants with and without automotive experience; that is, our study did not include participants who had never travelled by car. For this reason, differences between drivers and non-drivers can be taken at face value, that is, attributed to the act of controlling the vehicle. While physical driving experience did not influence the incidence or severity of motion sickness during virtual driving, it did affect participants’ head and torso movement before the onset motion sickness. The present study provides the first empirical evidence regarding the influence of long-term (i.e., decades) experience of physical driving experience on bodily movement and motion sickness while driving a virtual automobile.
